# Mono-(2-ethylhexyl) Phthalate Directly Alters the Expression of Leydig Cell Genes and CYP17 Lyase Activity in Cultured Rat Fetal Testis

**DOI:** 10.1371/journal.pone.0027172

**Published:** 2011-11-07

**Authors:** François Chauvigné, Simon Plummer, Laurianne Lesné, Jean-Pierre Cravedi, Nathalie Dejucq-Rainsford, Alexis Fostier, Bernard Jégou

**Affiliations:** 1 Inserm (Institut National de la Santé et de la Recherche Médicale), U625, IRSET (Institut de Recherche sur la Santé, l'Environnement et le Travail), Rennes, France; 2 CXR Biosciences Ltd, Dundee, United Kingdom; 3 INRA (Institut National de la Recherche Agronomique), UR1037 SCRIBE (Station Commune de Recherche en Ichtyophysiologie, Biodiversité et Environnement), Rennes, France; 4 INRA Toxalim, UMR 1331, Toulouse, France; Florida International University, United States of America

## Abstract

Exposure to phthalates in utero alters fetal rat testis gene expression and testosterone production, but much remains to be done to understand the mechanisms underlying the direct action of phthalate within the fetal testis.

We aimed to investigate the direct mechanisms of action of mono-(2-ethylhexyl) phthalate (MEHP) on the rat fetal testis, focusing on Leydig cell steroidogenesis in particular.

We used an in vitro system based on the culture for three days, with or without MEHP, of rat fetal testes obtained at 14.5 days post-coitum.

Exposure to MEHP led to a dose-dependent decrease in testosterone production. Moreover, the production of 5 alpha-dihydrotestosterone (5α-DHT) (−68%) and androstenedione (−54%) was also inhibited by 10 µM MEHP, whereas 17 alpha-hydroxyprogesterone (17α-OHP) production was found to increase (+41%). Testosterone synthesis was rescued by the addition of androstenedione but not by any of the other precursors used. Thus, the hormone data suggested that steroidogenesis was blocked at the level of the 17,20 lyase activity of the P450c17 enzyme (CYP17), converting 17α-OHP to androstenedione. The subsequent gene expression and protein levels supported this hypothesis. In addition to *Cyp17a1*, microarray analysis showed that several other genes important for testes development were affected by MEHP. These genes included those encoding insulin-like factor 3 (INSL3), which is involved in controlling testicular descent, and *Inha*, which encodes the alpha subunit of inhibin B.

These findings indicate that under in vitro conditions known to support normal differentiation of the fetal rat testis, the exposure to MEHP directly inhibits several important Leydig cell factors involved in testis function and that the *Cyp17a1* gene is a specific target to MEHP explaining the MEHP-induced suppression of steroidogenesis observed.

## Introduction

A number of endocrine disruptors (EDs) are known to be anti-androgenic [1], [2]. Several molecules exert their effect by inhibiting steroid synthesis within a “masculinization programming window” occurring between gestational day 15.5 and 19.5 in the rat [3], [4], of which are some phthalic acid esters widely used as additives in the manufacture of plastics [5]. Di-(2-ethylhexyl) phthalate (DEHP) is the most widely used phthalate [6]. Phthalates are of particular concern because epidemiological evidence suggests that the prenatal or neonatal exposure of boys to phthalates affects their masculinization (decreasing anogenital distance, [7]) and endocrine parameters [8]. Indeed, high di-n-butyl phthalate (DBP) and/or DEHP metabolite concentrations have been associated with low levels of human sex steroid hormones [9], [10].

The endocrine disrupting effects of phthalates have been extensively studied in the rat [2]. In this species, the male offspring of females exposed to phthalates during pregnancy were found to display reproductive abnormalities, such as hypospadias, cryptorchidism and malformations of the epididymis, vas deferens, seminal vesicles and prostate [3], [11]. These abnormalities are consistent with the phthalate-induced fetal inhibition of androgen production demonstrated by the administration of a number of phthalates during pregnancy in rats [2]. Furthermore, in an assay based on rat fetal testes in culture between 14.5 and 17.5 gestational days (GD), mono-(2-ethylhexyl) phthalate (MEHP), an active metabolite of DEHP, was found to inhibit testosterone production in a direct, time- and dose-dependent manner [12].

Phthalates do not bind the androgen receptor directly [13], [14]. Their effects on testosterone biosynthesis in the rat are thought to result from the inhibition of the expression of a number of enzymes involved in cholesterol uptake/transport and steroidogenesis [15], [16], [17], [18]. However, as these studies were performed *in utero*, it was not possible (1) to distinguish between direct effects and indirect effects involving metabolites; (2) to control the concentrations of phthalates actually acting on the fetal testis or (3) to exclude the possibility that phthalate-induced impairment of masculinization resulted from deficiencies in androgen action downstream from the testis, as opposed to changes in androgen production by the fetal testis itself.

The aim of the present studies was therefore first to identify the origin of the MEHP-induced androgen production inhibition, using an organotypic culture system for rat fetal gonads [19], which proved its usefulness for studying the direct endocrine disruptor-induced testicular dysfunction [12], [20]. Second, using this culture system, we aimed to determine whether the direct exposure of the rat testis to MEHP could induce other effects than on steroidogenesis and to identify them.

## Materials and Methods

### Animals and sample collection

Pregnant female Sprague-Dawley rats (from GERHM-Inserm, Rennes and Elevage Janvier, Le Genest Saint Isle, Laval, France) were anesthetized by an intraperitoneal injection of 50 mg/kg sodium pentobarbital (Sigma Chemicals, Saint-Quentin, France) on GD 14.5. The testes were aseptically removed from male fetuses under a binocular microscope and immediately explanted *in vitro*. Investigations were conducted according to the guiding principles for the use and care of laboratory animals and in compliance with French and European regulations on animal welfare ( Directive 86/609/CEE; agreement n°C35-238-19).

### Culture procedure

Testes were cultured on Millipore filters (pore size: 0.45 µm; Millipore, Molsheim, France), as previously described [12], [19], [20]. Briefly, each 14.5 GD fetal testis was placed on a filter floating on 0.5 ml of M199 medium (Invitrogen, Gibco, Eragny, France) supplemented with gentamicin (50 µg/ml; Life Technologies, Cergy-Pontoise France) and fungizone (2.5 µg/ml; Life Technologies, Cergy-Pontoise France) in 24-well tissue culture dishes, which were then incubated for 72 h at 37°C, under an atmosphere containing 5% CO2. Explanted testes were cultured with medium containing 1, 10 or 100 µM MEHP (Interchim SA, Montluçon, France). In a previous study where 10 µM of MEHP were added to the culture system, only 0.3% of them were present in the testicular fetal explants [12]; which corresponds to a concentration of about 2500 µg/L which is relevant to environmental exposures reported in humans. It is noteworthy that due to the fact that the culture procedure and analysis are tedious and very time-consuming, only the dose of 10 µM was selected when 5 alpha-dihydrotestosterone (DHT) was measured and/or when the origin of blockade of androgens production was investigated. The doses of 1 µM and 10 µM of MEHP were chosen for the microarray experiments. The MEHP stock solutions of 0.1, 1 and 10 mM were prepared in DMSO (Prolabo, Fontenay-sous-Bois, France), to give a final concentration of 1/100 (vol/vol) of DMSO in all conditions. Controls were incubated with 1/100 (vol/vol) DMSO. Half the volume of the medium was replaced daily, to keep the MEHP concentration constant. At the end of the culture period, the whole explant and the medium were frozen separately at −80°C, for further analysis.

### Testosterone, 5 alpha-dihydrotestosterone (5α-DHT) and 17 beta-estradiol (17β-E2) assays

After three days of culture, the medium was removed and stored at −80°C for hormone assays. Testosterone and its metabolites, 5 alpha-dihydrotestosterone (5α-DHT) and 17 beta-estradiol (17β-E2), were assayed with direct radioimmunoassay (RIA) kits (Diagnostic System Laboratories Inc., Beckman Coulter, Cergy-Pontoise, France). Each sample was assayed in duplicate, without prior extraction. Less than 0.1% cross-reaction with 5α-DHT or testosterone was observed in the testosterone and 5α-DHT assays, respectively. In the present assays, the coefficients of variation were less than 14% for testosterone, 9% for 5α -DHT and 12% for 17β-E2.

### 17 alpha-hydroxyprogesterone (17α-0HP) and androstenedione assays

17α-OHP and androstenedione levels were determined by competitive RIA [21]. Antibodies against 17-hydroxyprogesterone-3-(CMO)-BSA were obtained from Steranti (St Albans, UK) and antibodies against androstenedione-11alpha-succinyl-BSA were obtained from Dr Terqui (INRA, France). Determinations were carried out directly in 100 µl of culture medium without prior extraction. Each sample was assayed in duplicate. The sensitivity was 53 pg/ml for the 17α-OHP assay and 14 pg/ml for the androstenedione assay. Less than 1% cross-reaction was observed between testosterone and the antibodies against androstenedione and 17α-OHP, and about 3% cross-reaction was observed between progesterone and the antibody against 17α-OHP. In the present assays, the coefficients of variation were 5.7% and 6.3% for 17alpha-OHP and androstenedione, respectively.

### Supplementation of media with various steroids precursors during the culture period

In order to specify the enzymatic steps likely to be affected by MEHP for the synthesis of testosterone, several of its potential precursors, i.e. pregnenolone, 17α-OHP or androstenedione, were added at a concentration of 100 ng/ml (about 3.5×10^−7^ M), in the presence or absence of MEHP (10 µM). Such levels were 10 to 70-fold higher than those ones reported in blood of adult Sprague-Dawley male rat [22] and considered as reasonably close to the physiological endogenous concentrations for *in vitro* treatments of implants. After 72 h of culture, concentration values were determined in the medium for intermediary metabolites, i.e. 17α-OHP and androstenedione, and for testosterone as described above. The concentrations of ethanol used in our treatments (maximum 1%) had no effect on the testosterone production of the controls (n = 10) or testis morphology (data not shown). Finally, the effect of MEHP on 5alpha-reductase was investigated further by adding testosterone to the medium at a physiological concentration of 10^−7^ M in the presence or absence of MEHP (10 µM) and measuring 5α-DHT levels in the medium after 72 h culture.

### RNA labeling for microarray analysis

RNA was labeled with the Agilent Low-Input Linear Amplification Labeling Kit, according to the manufacturer's instructions (Agilent Technologies, Palo Alto, CA). Briefly, 25 ng of mRNA from one whole fetal testes treated with MEHP (1 µM or 10 µM) or from a control testis was used as a template for the synthesis of cDNA with the T7 primer, MMLV-RT and a master mix cDNA synthesis buffer containing 1× first strand buffer, 0.1 M dithiothreitol, 10 mM dNTPs, 1 IU MMLV-RT and 1 IU RNaseOUT. The MMLV-RT enzyme was inactivated to stop the reaction and the cDNA synthesis reaction mixture was divided in two. The cRNAs were labeled with the Agilent low-input fluorescence labeling kit, in a single cycle of linear amplification, according to the Agilent method (G414090050). Labeled cRNA was generated by adding cyanin-3 cytidine triphosphate (CTP) (Cy3) or cyanin-5 CTP (Cy5), T7 RNA polymerase, and transcription master mix. Labeling efficiency was checked with the microarray analysis program on a NanoDrop ND1000 spectrophotometer (Montchanin, USA), to ensure that only probes with sufficient Cy3/Cy5 incorporation were hybridized to the arrays.

### Microarray hybridization

Microarray hybridizations were performed with Agilent hybridization buffers and Agilent Whole Rat Genome 60-mer oligonucleotide arrays, with labeled RNA isolated from whole fetal testis explant cultures from a single MEHP-treated testes from each of three different litters compared with a control reference RNA sample (pooled RNA from 3 DMSO-treated fetal testes from three different litters) according to the Agilent method (G4140-90050). The use of a pool of RNA samples from control animals is the optimal method for detecting changes in gene expression related to MEHP treatment, because this approach minimizes the error due to inter-individual biological variation of gene expression in the control group. Hybridizations were performed in duplicate, to allow dye swapping. Thus, in one duplicate, the test RNA was labeled with Cy3 and the reference RNA was labeled with Cy5, and in the other, the labels were swapped (Cy5-labeled test and Cy-3-labeled reference RNAs). This duplication was required to control for the bias towards higher levels of Cy3 incorporation (as this molecule is smaller than Cy5) when these dyes are included in labeling reactions. Microarray analysis was performed with whole fetal testis RNA and Whole Rat Genome 4×44K 60mer oligonucleotide microarray kit (Agilent reference number: G4131F).

### Microarray data analysis and bioinformatics

Genes displaying significant (p<0.01) changes in expression in response to MEHP exposure were selected by Agilent feature extraction (v7.1) software, using an Agilent error model (Agilent Feature Extraction User Manual G2566-90012). Rosetta Resolver™ software (Rosetta Biosoftware, Kirkland, USA) was used to generate “signature” lists of genes displaying significant (p<0.01) regulation from replicate (n = 6 lots of 3 MEHP-treated testes * Cy3/cy5 and cy5/cy3) microarray hybridizations, by generating an error-weighted mean of fold-change (log ratio) values for the replicates. The “compare biosets” function in Resolver™ was used to compare signature lists from different fetal testis regions. Signature gene lists, including GenBank accession numbers and fold-change values, were also loaded into Ingenuity Pathways Analysis software for pathway analysis. The microarray data for this study is MIAME compliant and the raw data has been deposited in a MIAME compliant database: the NCBI Geo database (www.ncbi.nih.gov/geo/), under accession number GSE22218.

### Real-time quantitative RT-PCR analysis of gene expression

Six to 12 testes from the 10 µM MEHP treatment group or controls were pooled and total RNA was extracted with the RNeasy® Mini kit (Qiagen, Les Ulis, France) and treated with DNAse I. Total RNA concentration and integrity were checked by UV spectrophotometry. First-strand cDNAs were obtained from 2 µg of total RNA, by reverse transcription for 90 min at 42°C, this reaction being terminated by heating at 94°C for 2 min. Real-time quantitative RT-PCR (qPCR) amplification was performed in a final volume of 25 µl containing12.5 µl SYBR® Green qPCR master mix (Applied Biosystems France SA, Courtaboeuf, France), 1 µl of a 1/5 dilution of cDNA, and 0.5 µM gene-specific forward and reverse primers. The sequences were amplified in duplicate for each sample, in96-well plates, with the 7500 Real Time PCR sequence detection system (Applied Biosystems). The amplification protocol was as follows: initial denaturation and activation at 50°C for 2 min and 95°C for 10 min, followed by 40 cycles of 95°C for 15 s and 60°C for 1 min. A temperature-determining dissociation step was then carried out at 95°C for 15 s, 60°C for 15 s and 95°C for 15 s. For the normalization of cDNA loading, all samples were run in parallel, with the18S ribosomal protein (18S) gene used as a reference. Levels of 18S gene expression were similar in control and phthalate-exposed testes. A standard curve was generated for each primer pair, from two-fold serial dilutions of a pool of first-strand cDNA templates from all samples. Standard curves showed the cycle threshold (Ct) value to be a function of the logarithm of the number of copies generated, defined arbitrarily as 1 copy for the standard cDNA pool. The genes involved in steroidogenesis which were investigated were: *Cyp11a1*, encoding the enzyme responsible for converting cholesterol to pregnenolone, *Hsd3b1*, encoding 3-beta-hydroxysteroid dehydrogenase isomerase 1 (3βHSD), which generates progesterone from pregnenolone, *Cyp17a1*, encoding the enzyme with 17α-hydroxylase activity responsible for converting progesterone to 17-hydroxyprogesterone and 17-hydroxy-progesterone to androstenedione *via* its 17,20 lyase activity. Furthermore, the gene *Star* encoding a key cholesterol transport protein was also assessed. The forward and reverse primers were as follows:

*Star* forward-CTGCTAGACCAGCCCATGGAC, *Star* reverse-TGATTTCCTTGACATTTGGGTTCC;

*Cyp11a1* forward-GGAGGAGATCGTGGACCCTGA, *Cyp11a1* reverse-TGGAGGCATGTTGAGCATGG;

*Hsd3b1* forward-AGCAAAAAGATGGCCGAGAA, *Hsd3b1* reverse-GGCACAAGTATGCAATGTGCC;

*Cyp17a1* forward-TGGCTTTCCTGGTGCACAATC, *Cyp17a1* reverse-TGAAAGTTGGTGTTCGGCTGAAG;

### Protein extraction and western blotting

For protein extraction, we lysed a pool of eight testes per treatment (MEHP samples vs controls) in RIPA buffer containing a mixture of protease inhibitors (Sigma), 50 mM Tris pH 8, 150 mM NaCl, 1% Nonidet P-40, 0.5% sodium deoxycholate and 0.1% SDS. Total protein extract (20 µg) was denatured in Laemmli buffer at 95°C and separated by SDS-PAGE. Proteins were transferred onto PVDF membranes and stained with Ponceau Red. The membranes were then hybridized with 1/100 anti-p450c17 (sc-46081), 1/500 anti-cytochrome b5 (sc-33174) or 1/500 anti-GADPH (sc-25778) (Santa-Cruz Biotechnology, Heidelberg, Germany) antibodies, overnight at 4°C. Horseradish peroxidase-conjugated secondary antibodies (donkey anti-goat, 705-035-147, Jackson ImmunoResearch, Suffolk, UK and donkey anti-rabbit, NA9340V, Amersham, Les Ulis, France) were added and the membrane was incubated for 1 hour at room temperature. Antibody binding was then detected by processing for 2 minutes with the ECL™ Plus Western Blotting Kit (Amersham). Membranes were then immediately placed against Kodak Biomax MR film for 1 to 10 minutes. The films were scanned and the intensity of the bands was determined with Quantity One software (Biorad, Marnes la Coquette, France).

### Statistical analysis

Wilcoxon Mann-Whitney tests were performed on unpaired data. For the dose-response experiments, we carried out a non parametric analysis of variance (ANOVA). We used SAS/STAT software (version 9.1; SAS institute Inc., Cary, NC, USA) for all statistical analyses.

## Results

### MEHP decreases testosterone and 5α-DHT levels

**Figure 1:**
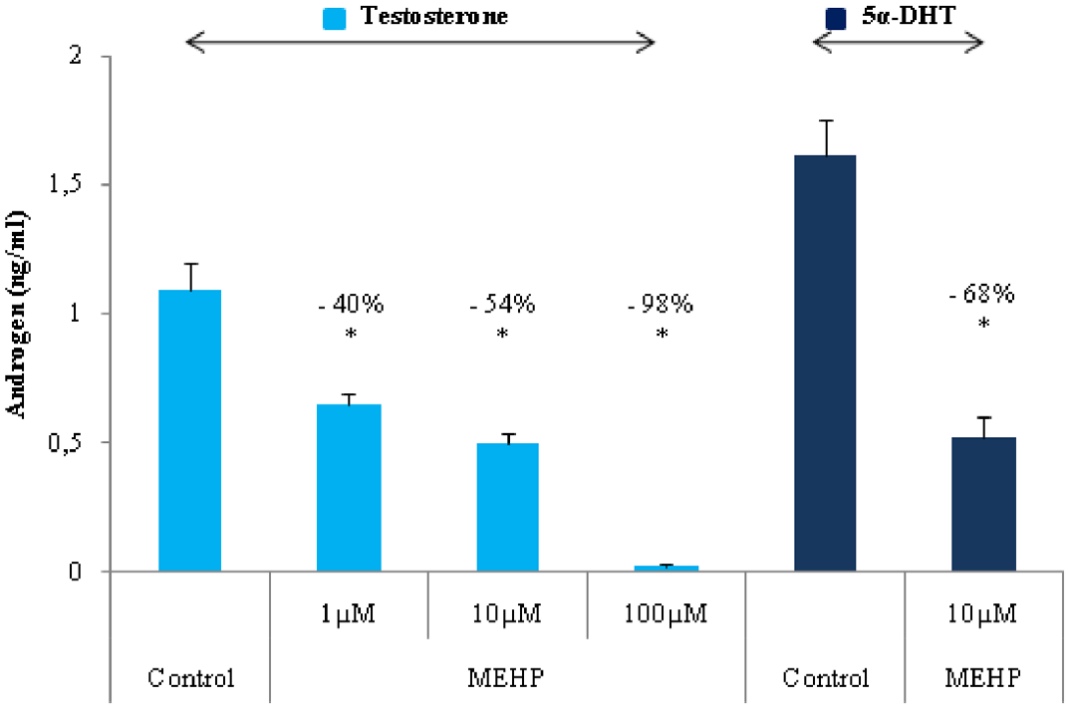
MEHP inhibits the production of testosterone and 5α-DHT by the fetal testis. Effects of MEHP on the production of testosterone and 5α-DHT by fetal rat testes cultured for 72 h beginning on GD14.5. Testosterone and 5α-DHT were determined with specific RIAs. Control testes were cultured in medium containing DMSO, whereas the treated testes were cultured in medium containing 1, 10 or 100 µM MEHP diluted in DMSO. The testes of different fetuses from different dams were selected at random. Values are means +/− SEM of 10 testes from the fetuses of 2 litters in 3 independent experiments. The number indicates the percentage decrease relative to the corresponding control. * p<0.05 in Wilcoxon Mann-Whitney tests comparing treated and control testes in the 5alpha-DHT assay * p<0.05 in non parametric *ANOVA for the MEHP dose-response testosterone assay*.

The incubation of fetal testis explants with MEHP resulted in the dose-dependent inhibition of testosterone production (Fig. 1). 5α-DHT is the principal androgen active in the development of a number of tissues/organs of the male genital tract [23]. We therefore determined its concentration in the medium. For the controls, the concentration of 5α-DHT in the medium was significantly higher than that of testosterone itself (Fig. 1), demonstrating that the steroid-5α-reductase enzyme responsible for converting testosterone to 5α-DHT was active in the explanted testes over the culture period. MEHP exposure (10 µM) resulted in markedly lower concentrations of 5α-DHT, indicating that the phthalate-induced decrease in testosterone levels was unlikely to be due to an increase in its 5α reduction rate (Fig. 1). 17β-E2 concentrations in the culture medium were also determined, to investigate the possibility of an MEHP-induced change in the conversion of testosterone to 17β-E2 by aromatase (P450arom). However, 17β-E2 levels remained below the detection limit of the radioimmunoassay (RIA), suggesting that aromatase may be produced in only small amounts or inactive within the fetal testis at GD 14.5–17.5 (data not shown).

### MEHP blocks the conversion of 17 alpha-hydroxyprogesterone (17α-OHP) to androstenedione

**Figure 2:**
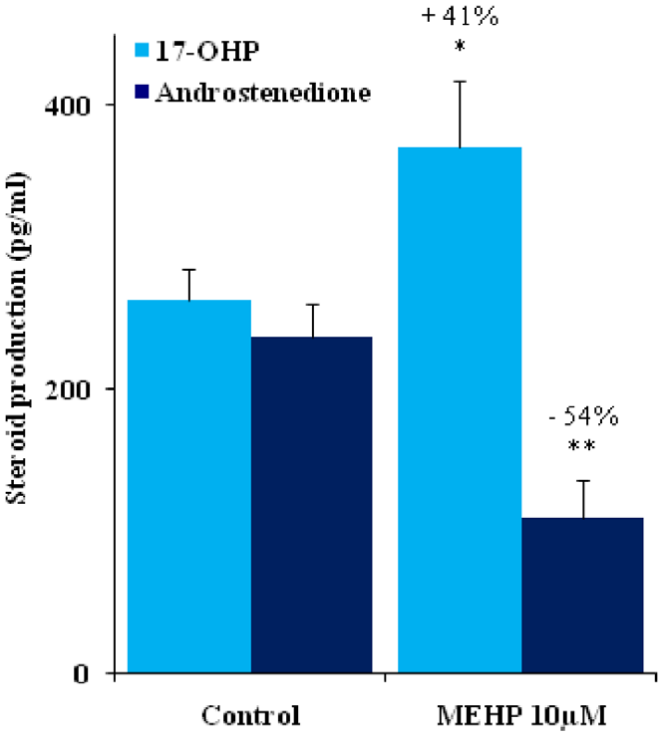
17-hydroxyprogesterone (17-OHP) production is increased whereas androstenedione production is decreased by exposure of the fetal testes to MEHP. Effects of 10 µM MEHP on 17-OHP and androstenedione secretion by fetal rat testes cultured for 72 h, beginning at GD14.5. We determined 17-OHP and androstenedione concentrations with specific RIAs. Values are means +/−SEM of 8 (17-OHP) and 7 (androstenedione) testes from fetuses of 2 different litters in 2 independent experiments. The numbers indicate the percentage decrease or increase relative to the corresponding control. * p<0.05 and ** p<0.01 in *Wilcoxon Mann-Whitney tests comparing treated and control testes*.

Androstenedione and 17α-OHP concentrations were determined, for investigation of the Δ4 steroidogenic pathway [2]. MEHP exerted its inhibitory effects on testosterone production. *In vitro* MEHP treatment (10 µM) decreased the concentration of androstenedione, the immediate upstream Δ4 steroid precursor of testosterone, to a similar extent to that of testosterone (−53.7% in MEHP-treated testes versus control testes; p<0.01) (Fig. 2). By contrast, 17α-OHP, the substrate of 17,20-lyase for androstenedione synthesis, accumulated in the medium after MEHP exposure (+40.9%; p<0.05) (Fig. 2).

For confirmation that MEHP blocked mainly the conversion of 17alpha-OHP to androstenedione, we analyzed 17α-OHP, androstenedione, testosterone and 5α-DHT concentrations after three days of culture in medium supplemented with pregnenolone, 17α-OHP, androstenedione or testosterone.

**Figure 3:**
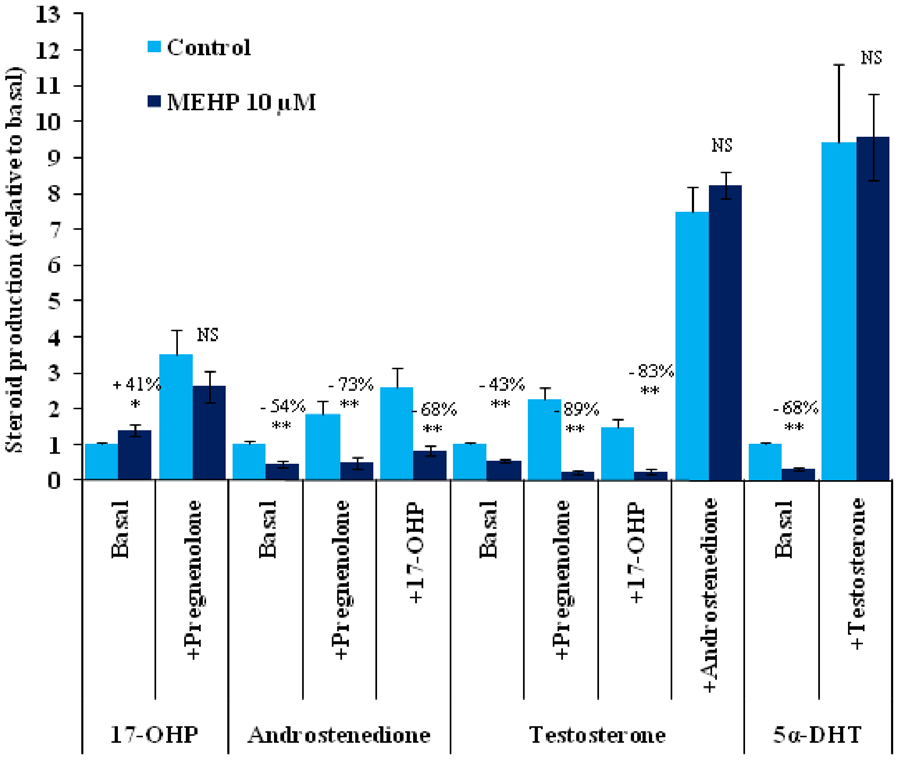
Supplementation of the medium with testosterone and testosterone precursors reveals that only the 17,20 lyase activity of CYP17 is affected by MEHP treatment. Effect of 3.5×10^−7^ M pregnenolone (+Pregnenolone), 17-OHP (+17-OHP) androstenedione (+Androstenedione) or 10^−7^ M testosterone (+Testosterone) in the presence or absence of 10 µM MEHP on 17-OHP, androstenedione, testosterone and 5α-DHT secretion *in vitro*. Data are presented relative to the corresponding control (Basal). Values are means +/−SEM of 7 testes from fetuses taken from 2 litters in 3 independent experiments. * p<0.05 and ** p<0.01 in *Wilcoxon Mann-Whitney tests comparing treated and control testes; NS: not significant*.

Consistent with the observed blockade of the conversion of 17α-OHP to androstenedione, pregnenolone supplementation led to a significant increase in 17α-OHP concentration in both control and MEHP-treated testes (Fig. 3). Thus, neither 3 beta-hydroxysteroid deshydrogenase (3β-HSD) nor CYP17 17α-hydroxylase activity was inhibited by MEHP. Our data also confirmed that the conversion of 17α-OHP to androstenedione by the 17,20 lyase activity of CYP17 was indeed inhibited by MEHP, as neither pregnenolone nor 17α-OHP rescued androstenedione levels in MEHP-treated testes. By contrast, the supplementation of controls with these agents caused an increase in androstenedione levels (Fig. 3). The specificity of CYP17 lyase activity inhibition by MEHP was further demonstrated by the demonstration that supplementation with pregnenolone and 17α-OHP did not increase testosterone synthesis in testes exposed to MEHP. However, androstenedione supplementation rescued testosterone production to levels similar to those in control testes (Fig. 3), indicating that 17 beta- hydroxysteroid deshydrogenase (17β-HSD) activity was unaffected by MEHP treatment.

Finally, the addition of testosterone to the medium resulted in an increase in 5α-DHT concentration similar to that observed for androstenedione-induced testosterone production, indicating that the steroid-5α-reductase enzyme was highly active in cultured fetal rat testis and that MEHP did not directly inhibit the production or activity of this enzyme (Fig. 3).

### Direct effects of MEHP on fetal testis gene expression

We then tried to identify the genes most likely to be directly affected by MEHP. Microarray analysis of MEHP-exposed and non exposed fetal gonads revealed differential expression of genes involved in lipid and cholesterol metabolism and transport, steroidogenesis, redox homeostasis and testis descent and development (Table 1).

**Table 1:** Genes involved in testicular development and functions affected in fetal testis explants exposed to MEHP.

		Fold-change in expression	
Accession number	Gene	MEHP 1 µM	MEHP 10 µM
**LIPID METABOLISM AND TRANSPORT**			
NM_138828	*Apoe*	**1.96** **	**1.50** **
NM_022512	*Acads*	NS	**1.33** **
NM_017340	*Acox1*	NS	**1.27** *
NM_012839	*Cycs*	NS	*−2.05* **
NM_001169113.1	*Lrpap1*	NS	*−2.71* **
NM_024162	*Fabp3*	NS	*−4.87* **
**CHOLESTEROL METABOLISM AND TRANSPORT**			
NM_031558	*Star*	**3.46** **	NS
NM_031541	*Scarb1*	**2.07** **	NS
NM_017268	*Hmgcs1*	**1.82** **	*−1.48* **
NM_031840	*Fdps*	NS	*−2.86* **
NM_012941	*Cyp51*	NS	*−2.89* **
**STEROIDOGENESIS**			
NM-057137	*Ebp*	**2.92** **	**2.16** **
NM_017126	*Fdx1*	NS	*−2.73* **
NM_012753	*Cyp17a1*	*−2.24* **	*−4.20* **
**REDOX HOMEOSTASIS**			
NM_030826	*Gpx1*	*−1.24* *	*−2.35* **
NM_001106840.1	*Gsta4*	*−1.67* *	*−3.10* **
**TESTIS DESCENT AND DEVELOPMENT**			
NM_017169	*Prdx2*	NS	*−1.75* **
NM_013177	*Got2*	NS	*−3.30* **
NM_031819	*Fat1*	NS	*−1.27* **
NM_175761	*Hspca*	NS	*−2.30* *
NM_012590	*Inha*	*−1.66* **	*−1.80* **
NM_031140	*Vim*	*−1.26* **	*−2.14* *
NM_053680	*Insl3*	*−3.55* **	*−2.95* **
NM_017236	*Pbp*	*−1.58* **	*−4.00* **

Fold-change values expressed relative to time-matched untreated controls, with reciprocal transformation from expression ratios. NS: no significant change in gene expressions. Values in italics indicate a down-regulation and values in bold indicate an upregulation.

*p value<0.01 and

**p<0.001.

MEHP (10 µM) treatment also modified the expression patterns of genes encoding factors important for lipid homeostasis - apolipoprotein E (APOE), acyl-coenzyme A dehydrogenase (ACADS) and acyl-coenzyme A oxidase 1 (ACOX1) were upregulated- whereas genes encoding for cytochrome c (CYCS), low-density lipoprotein receptor-related protein-associated protein 1 (LRPAP1) and fatty acid-binding protein 3 (FABP3) were downregulated.

At the lowest concentration tested (1 µM), MEHP upregulated expression of the genes encoding scavenger receptor class B member 1 (SR-B1) and steroidogenic acute regulatory protein (STAR), responsible for the entry of cholesterol into Leydig cells and its transfer to mitochondria, where it is metabolized, respectively. However, 10 µM MEHP had no significant effect on these genes. MEHP upregulated the gene encoding for 3hydroxy-3-methylglutaryl-coenzyme A synthase 1 (HMGCS1) when added to the medium at a concentration of 1 µM, but inhibited it at a concentration of 10 µM MEHP. The genes, encoding farnesyl diphosphate synthase (FDPS) and cytochrome P450 family 51 (CYP51), which are involved in cholesterol biosynthesis, were both downregulated by 10 µM MEHP.

Within the group of genes encoding steroidogenic enzymes, only the expression of *Cyp17a1*, encoding the 17hydroxylase/17,20-lyase enzyme responsible for converting progesterone to androstenedione, was significantly inhibited (Table 1), in a concentration-dependent manner. That expression of *Cyp17a1* may be inhibited by MEHP was confirmed by real-time quantitative RT-PCR. Other genes encoding proteins involved in steroidogenesis regulation, such as emopamil-binding protein (sterol isomerase) (EBP) and ferredoxin 1 (FDX1), were significantly up- and down-regulated, respectively (Table 1).

Two genes encoding proteins involved in redox homeostasis, which are important for the regulation of steroidogenic enzyme activities - glutathione peroxidase 1 (GPX1) and glutathione S-transferase alpha 4 (GSTA4) - were found to be downregulated in a dose-dependent manner by MEHP.

Genes involved in testis development for which expression was modulated by MEHP exposure included those encoding peroxiredoxin 2 (PRDX2), glutamic-oxaloacetic transaminase 2 (GOT2), FAT tumor suppressor homolog 1 (FAT1), heat shock protein 90 alpha class A member (HSPCA), inhibin alpha (INHA), vimentin (VIM), insulin-like factor 3 peptide (INSL3) and phosphatidylethanolamine-binding protein 1 (PBP). All these genes were downregulated by MEHP exposure in a dose-dependent manner. Of particular importance, MEHP affected the expression of the *Insl3* gene, which is known to be expressed by Leydig cells and is involved in gubernacular ligament development and testicular descent [24], [25]. Of note also is that the *Inha* gene, encoding the alpha subunit of Inhibin B and which is known to be expressed in both Leydig and Sertoli cells during fetal life [26] was also found to be down-regulated by MEHP.

**Figure 4:**
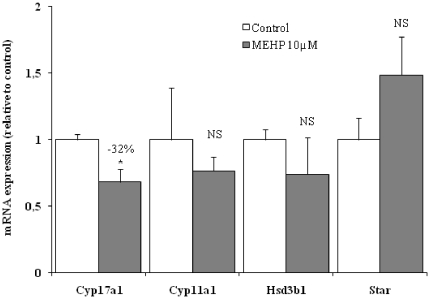
Expression of a number of genes involved in cholesterol transport and steroidogenesis, as determined by real-time quantitative PCR. *18 S* transcript levels were used as a reference, and relative transcript levels were calculated as a fold-change relative to control. The names of the genes investigated are cytochrome P450, family 17, subfamily A, polypepetide 1 (*Cyp17a1*), cytochrome P450, family 11, subfamily A, polypepetide 1 (*Cyp11a1*), 3 beta- and steroid delta-isomerase 1 (*Hsd3b1*), Steroidogenic acute regulatory protein (*Star*). Data are presented as means ± SEM for various cDNAs synthesized from the mRNA of 6 to 12 testes from the fetuses of 2 litters in 3 or 4 independent experiments. The number indicates the percentage decrease relative to the control. ^*^ p<0.05 in *Wilcoxon Mann-Whitney tests comparing treated and control to testes; NS: not significant*.

For validation of the microarray analysis, RT-PCR was carried out on a number of genes involved in steroid production and cholesterol transport. Consistent with the results of microarray analysis, the *Cyp17a1* gene was the only gene encoding a steroidogenic enzyme displaying lower levels of expression (−32%; p<0.05) in MEHP-exposed testes than in unexposed organs. Furthermore, the RT-PCR measurements also confirmed that *Star* expression in MEHP-treated testes tended to be higher than in controls (Fig. 4).

### MEHP decreases CYP17 protein levels

**Figure 5:**
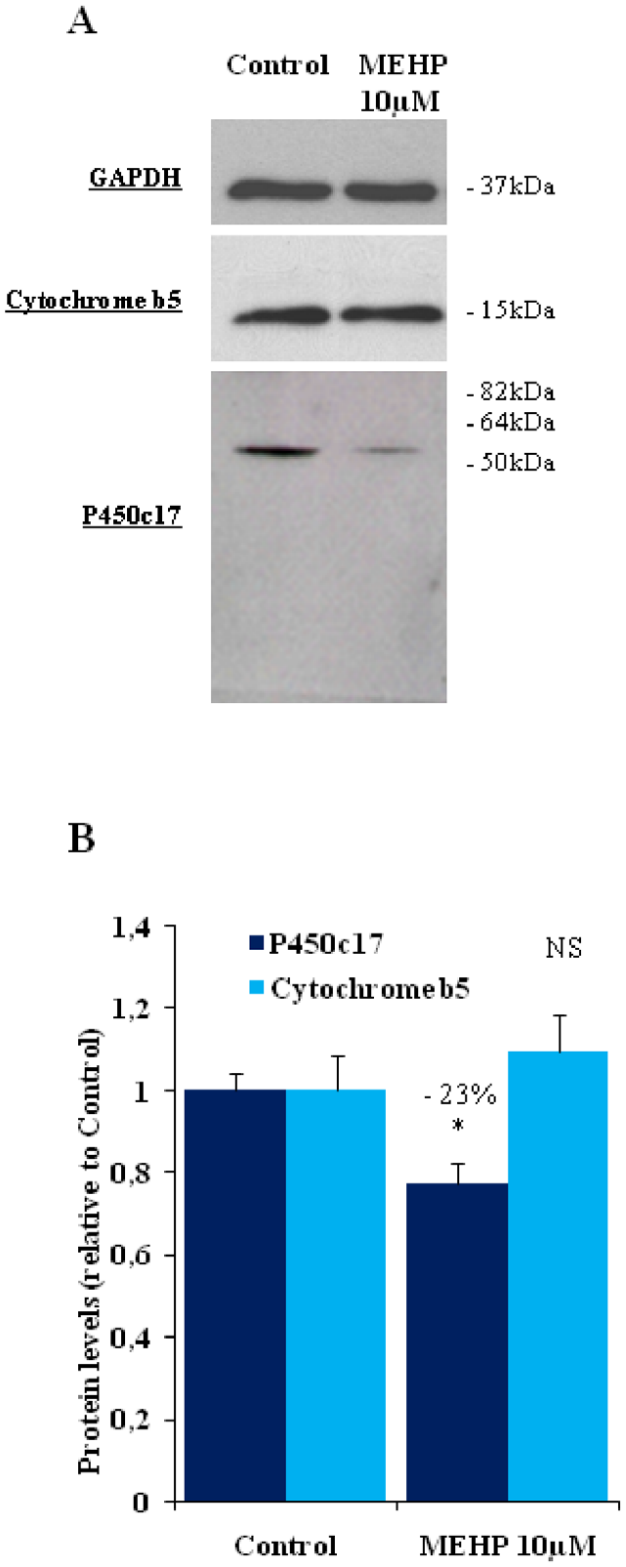
Western blot analysis of P450c17 and Cytochrome b5 protein levels after MEHP exposure. A) Representative Western blot of protein extracts (20 µg) from a pool of 8 control testes or testes exposed to 10 µM MEHP. Blots were incubated with anti-p450c17, anti-Cytochrome b5 and anti-GAPDH antibodies, to control for equal loading. The apparent molecular masses (kDa) are indicated on the left. B) Quantification of band intensity on three blots, carried out with Quantity One software (Biorad). Data are expressed in arbitrary units relative to the corresponding control. The number indicates the percentage decrease relative to control. * *p<0.05 in Wilcoxon Mann-Whitney tests comparing treated and control testes.* *NS: not significant*.

Consistent with the corresponding levels of mRNA, testis CYP17 protein concentration was also significantly decreased (−23%; P<0.05) by MEHP treatment, whereas no significant change was observed for the cytochrome b5 protein (Fig. 5A&B).

## Discussion

Changes in the production or action of testosterone are known to cause a number of disorders, such as hypospadias, cryptorchidism and abnormally low levels of sperm production [1], [2], [4], [27]. Endocrine disruptors (EDs) have received considerable attention over the last 15 years as they have been experimentally shown to participate in these disorders, which are increasing in incidence in humans [28], [29], [30].

The EDs known to generate these disorders include a number of antagonists of testosterone action and inhibitors of testosterone synthesis [1], including the industrial chemicals phthalate esters [31].

Antiandrogenic effects of DBP and DEHP have been demonstrated in the rat for doses exceeding 100 to 250 mg/kg/day administered *in utero* [2], [32], [33]. Although essential to the understanding of the toxicity of phthalates, these experiments have one limitation is that they do not allow to conclude whether these effects on the fetal testis were indirect (i.e. via effects on the mother), direct, or both. In this context, the use of *in vitro* approaches is essential.

We recently demonstrated, in an *in vitro* fetal gonad assay that MEHP, the bioactive metabolite of DEHP (but not DEHP itself) inhibited rat Leydig cell testosterone production [12]. This inhibition of testosterone production (i) occurred in the absence of any change in Leydig cell number, (ii) was time- and dose-dependent, (iii) occurred while the fetal testes do not have the ability to metabolize DEHP or MEHP and (iv) at concentrations relevant to human exposure [12]. Furthermore this inhibition appeared to be independent of the other negative effects observed in the germ cells [12]. The results presented here confirm that MEHP inhibits testosterone production in our rat fetal gonad assay and also reveal that 5alpha-DHT production is strongly decreased as a consequence of testosterone synthesis inhibition.

The complementary approaches used in this study to explore the origin of the MEHP-induced inhibition of androgen production by the fetal testis indicated that CYP17/*Cyp17a1* was a target of MEHP. *In vivo/in utero*, phthalates have been shown to have broadly inhibitory effects on the expression of genes encoding steroidogenic enzymes. Thus, in addition to *Cyp17a1*, the genes encoding cytochrome P450scc and 3beta-HSD have also been shown to be inhibited by phthalates [16], [17], [34], [35], [36]. The doses of phthalates used in these experiments may also induce changes through systemic effects on the pregnant mother or the fetus itself. Our results clearly indicate that CYP17 is the direct target of MEHP in fetal rat testes.

We demonstrate that MEHP specifically blocks the 17,20 lyase activity of CYP17. The two activities of CYP17 (i.e. lyase and hydroxylase) are regulated by the ratio of hormones to their substrates [37] and by redox partners.

Our microarray analyses revealed that the genes encoding oxidative stress proteins such as glutathione peroxidase 1 (GPX1) and glutathione S-transferase alpha type 4 (GSTA4) were downregulated by MEHP, possibly as a consequence of testosterone inhibition [38]. GPX1 and GSTA are essential for activation of the 17α-hydroxylase and 17,20 lyase activities of the enzyme through electrostatic interactions with their cofactors, principally cytochrome b5 and P450 oxidoreductase (POR) [39]. In contrast to the reported effects of phthalate exposure *in utero* [40], the genes encoding POR and cytochrome b5 were not found here to be downregulated in fetal testis explants. These findings were confirmed, for *Cyb5a*, by RT-PCR (data not shown) and western blotting. Thus, the phthalate-induced inhibition of CYP17 does not result from direct inhibition of the interaction of cytochrome b5 with CYP17. In this study, MEHP decreased expression of the gene encoding ferredoxin 1 (FDX1), a protein that regulates the first enzymatic reaction of the steroidogenic pathway, catalyzed by cytochrome P450scc [41]. *Fdx1* was also found to be downregulated by phthalates *in utero*, potentially accounting, at least in part, for their anti-androgenic action [17], [40]. However, we show here that the addition of pregnenolone *in vitro* does not rescue testosterone levels, suggesting that the effects of MEHP on *Fdx1* gene expression are unlikely to be a key element in its effects on androgen production *in vitro*.

In contrast to published findings for *in utero* experiments [15]–[18], [40], our *in vitro* assay showed the upregulation of three genes encoding proteins involved in cholesterol synthesis and transport the steroidogenic acute regulatory protein (StAR), the hydroxy-3-methylglutaryl-coenzyme A (HMGCS1), and scavenger receptor class B isoform 1, (SR-B1) by the lowest concentration (1 µM) of MEHP used, in fetal testes explants. However, the induction of *Star* and *Scarb1* gene expression observed with 1 µM MEHP were not observed with 10 µM MEHP. In 40-day-old rat immature Leydig cells, *Star* expression was shown to be inhibited *in vitro* by a high dose of MEHP (250 µM) [42]. Accordingly, a recent study has shown that not only *Cyp17a1* but also *Star* expression were inhibited in MEHP-pretreated mouse Leydig tumor cells MA-10 stimulated by LH [43]. We also observed an increase in the abundance of mRNA for genes involved in lipid and cholesterol synthesis, which may regulate testosterone production in rat Leydig cells: apolipoprotein E (*Apoe* [44], [45]), *Acads* [46] and the sterol 8-isomerase (*Ebp* [47]). As SR-B1, StAR, HMGCS1, APOE and EBP are all involved at various levels of the steroidogenic cascade, it is possible that the MEHP-induced stimulation of the expression of their genes observed here at the lowest dose of MEHP (1 µM) corresponds to a negative feedback compensation mechanism counterbalancing the MEHP-induced inhibition of *Cyp17a1* gene expression. The antiandrogen flutamide has been shown to have similar effects on these genes in rats treated *in vivo* [48]. The observed differences between our previous *in utero* experiments and this *in vitro* analysis, particularly in terms of the expression of the *Star* and *Scarb1* genes, may be accounted for by the absence of maternal effects of MEHP in this *in vitro* system and/or differences in the doses used [12].

Expression of the gene encoding phosphatidylethanolamine-binding protein 1 (PBP) was inhibited, in a dose-dependent manner, by MEHP. This may be an indirect consequence of the MEHP-induced decrease in testosterone production, as *Pbp* levels have also been shown to be decreased by castration in the rat [49] and by exposure to other antiandrogenic agents, such as prochloraz or flutamide, in castrated rats [50]. The microarray analysis revealed that expression of a gene encoding the insulin-like factor 3 peptide (INSL3) and essential for testis positioning was directly inhibited by MEHP, confirming previous observations following the administration of DBP and/or DEHP to rats and mice *in vivo* [15], [17], [51] or the exposure of mouse and rat fetal Leydig cells [52]. Moreover, the gene *Inha* was inhibited by MEHP, supporting the hypothesis that this gene is a target of phthalates, consistent with data from *in utero* studies [17], [40]. The *Inha* gene is expressed in both Leydig cells and Sertoli cells, but the phthalate-induced downregulation of this gene occurs only in interstitial (Leydig) cells [17]. It was proposed that phthalate inhibits Inhibin secretion by Leydig cells, thereby modifying Sertoli cell development [40].

Because an organotypic culture system was used in this study, it cannot be excluded that the effects observed may not be direct on Leydig cells but mediated by another/others cell types(s) of the testis. However, the fact that (*i*) the deleterious effects of MEHP chronogically preceded that on germ cells and on Sertoli cells [12], and that (*ii*) MEHP-induced suppression of testosterone production was observed when isolated Leydig cells and the mouse MA-10 cells were used [43], [53] clearly show that MEHP can directly target the Leydig cells. Of not also that published data show that most genes with expression patterns altered by exposure to DBP exposure *in utero* are expressed in the interstitial (Leydig cell) compartment, with considerably fewer of the genes expressed in the chords (Sertoli and germ cells) being altered [17]. Other experiments evidenced the rat Leydig cell as being a target to DEHP after *in utero* or post-natal exposures [14], [27], [33]. Here we demonstrate that MEHP, a widespread endocrine disruptor, blocks androgen production by specifically targeting the 17,20 lyase activity of CYP17.

In conclusion, our data confirm that several important functions of the fetal Leydig cells can be altered by MEHP and provide evidence that its effects result from its direct effects on the fetal testis and that the direct effects of MEHP on the production of both androgens and INSL3 by Leydig cells must probably contribute to the dysgenic effects of this phthalate on the reproductive organs of the male rat and their development.
